# Halotolerant bacteria in the São Paulo Zoo composting process and their hydrolases and bioproducts

**DOI:** 10.1590/S1517-838246220130316

**Published:** 2015-06-01

**Authors:** Lilian C.G. Oliveira, Patricia Locosque Ramos, Alyne Marem, Marcia Y. Kondo, Rafael C.S. Rocha, Thiago Bertolini, Marghuel A.V. Silveira, João Batista da Cruz, Suzan Pantaroto de Vasconcellos, Luiz Juliano, Debora N. Okamoto

**Affiliations:** 1 Universidade Federal de São Paulo, Departamento de Biofísica, Escola Paulista de Medicina, Universidade Federal de São Paulo, São Paulo, SP, Brasil, Departamento de Biofísica, Escola Paulista de Medicina, Universidade Federal de São Paulo, São Paulo, SP, Brazil.; 2 Universidade Federal de São Paulo, Departamento de Ciências Biológicas, Universidade Federal de São Paulo, Diadema, SP, Brasil, Departamento de Ciências Biológicas, Universidade Federal de São Paulo, Diadema, SP, Brazil.; 3 Laboratório de Microbiologia Aplicada, Fundação Parque Zoológico de São Paulo, São Paulo, SP, Brasil, Laboratório de Microbiologia Aplicada, Fundação Parque Zoológico de São Paulo, São Paulo, SP, Brazil.; 4 Koppert Biological Systems, Itapetininga, SP, Brasil, Koppert Biological Systems, Itapetininga, SP, Brazil.

**Keywords:** halophilic, protease, lipase, amylase, cellulase

## Abstract

Halophilic microorganisms are able to grow in the presence of salt and are also excellent source of enzymes and biotechnological products, such as exopolysaccharides (EPSs) and polyhydroxyalkanoates (PHAs). Salt-tolerant bacteria were screened in the Organic Composting Production Unit (OCPU) of São Paulo Zoological Park Foundation, which processes 4 ton/day of organic residues including plant matter from the Atlantic Rain Forest, animal manure and carcasses and mud from water treatment. Among the screened microorganisms, eight halotolerant bacteria grew at NaCl concentrations up to 4 M. These cultures were classified based on phylogenetic characteristics and comparative partial 16S rRNA gene sequence analysis as belonging to the genera *Staphylococcus*, *Bacillus* and *Brevibacterium*. The results of this study describe the ability of these halotolerant bacteria to produce some classes of hydrolases, namely, lipases, proteases, amylases and cellulases, and biopolymers. The strain characterized as of *Brevibacterium avium* presented cellulase and amylase activities up to 4 M NaCl and also produced EPSs and PHAs. These results indicate the biotechnological potential of certain microorganisms recovered from the composting process, including halotolerant species, which have the ability to produce enzymes and biopolymers, offering new perspectives for environmental and industrial applications.

## Introduction

The biocatalysts required in several industrial processes exhibit optimal activities at high ranges of salt concentration, pH and temperature. Halophiles are excellent sources of such enzymes and are found in nearly all major microbial clades, including prokaryotic (Bacteria and Archaea) and eukaryotic forms; two categories have been defined: halotolerant microorganisms that are adapted in live at high salinity, and halophiles that require salinity for growth. Halotolerant species tend to live in areas of salinity, such as hypersaline lakes, coastal dunes, saline deserts and salt seas (Ventosa and Nieto, 1995).

Halophilic enzymes perform the same enzyme function as their non-halophilic counterparts but require 1–4 M salt concentrations for their full activity and stability. In addition, these enzymes typically demonstrate a large excess of acidic amino acids compared to basic residues (Enache and Kamekura, 2010).

Proteases constitute approximately 66% of the total enzymes employed in biotechnological and commercial processes (Gupta *et al.*, 2002), and the moderately halophilic aerobic bacteria of genera *Bacillus*, *Pseudomonas*, *Halomonas* and *Serratia* are important sources of proteases (Ventosa *et al.*, 1998). Amylases are extensively studied due to their potential application in the food, detergent, paper and pharmaceutical industries, representing approximately 25% of the total enzymes in the industrial market. The extracellular production of β-amylase by halophilic *Halobacillus* sp. LY9 and of two α-amylases from *Chromohalobacter* sp. has been reported (Li and Yu, 2011; Prakash *et al.*, 2009). Cellulases also have industrial application, including the generation of bioethanol and in the textile industry, and a halotolerant cellulase was characterized in a soil metagenome analysis (Voget *et al.*, 2006). Lipolytic enzymes are of particular industrial interest, and their identification in halophilic bacteria has been reported and recently reviewed (Gomez *et al.*, 2012). Exopolysaccharides (EPSs) and polyhydroxyalkanoates (PHAs) are biotechnological products that were identified and produced from halophilic/halotolerant microorganisms (Legat *et al.*, 2010; Litchfield, 2012).

In this sense, the Organic Composting Production Unit (OCPU) of SPZPF is a potential source of microorganisms, as demonstrated by an OCPU metagenomic analysis, which revealed a diversity of biomass degradation functions and organisms (Martins *et al.*, 2013). The composting process is predominantly aerobic, with organic residues being degraded by microorganisms, generating a humus-like material. In recent years, composting has attracted attention as a viable and environmentally adequate alternative for the treatment of organic waste. The initial phase of composting is thought to be the most dynamic part of the process and is characterized by a rapid increase in temperature, a large change in pH, and the degradation of simple organic compounds (Schloss *et al.*, 2003). A detailed comparison of the bacterial diversity from different composting plants revealed a large difference at both the species and strain levels (Partanen *et al.*, 2010).

This paper reports the screening of the composting process of OCPU at SPZPF for bacteria in the presence of a range of NaCl concentrations and also the evaluation of their potential for the production hydrolases and biopolymers. To date, the microbial diversity of this ecosystem has not been explored, particularly with regard to the screening of halotolerant microorganisms.

## Material and Methods

### Bacterial strains and isolation of DNA

#### Composting process

**Figure 1:**
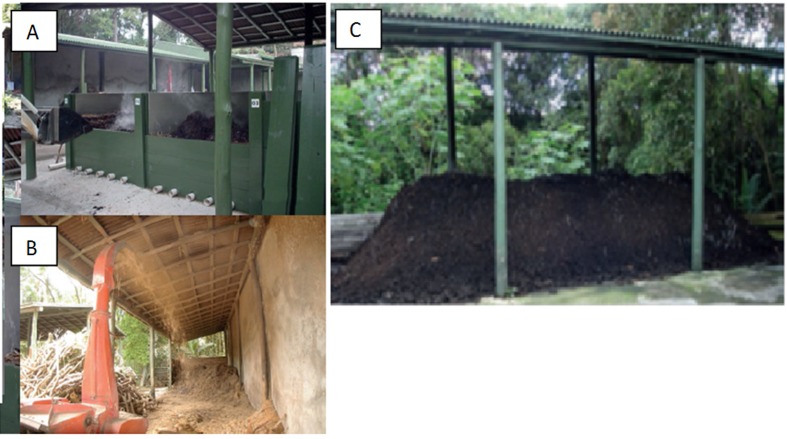
Partial view of the Organic Composting Production Unit (OCPU) of São Paulo Zoological Park Foundation. (A) Aerobic composting process in the thermophilic phase; (B) grinding of plant matter from the Atlantic Rain Forest; (C) maturation phase of the composting process.

The composting process was conducted in the SPZPF OCPU in 2.5 × 2.0 × 1.6 m (length×width×height) cells, as shown in Figure 1. The piles were formed by organic residues including food, droppings and excreta, the beds of native and exotic wild animals, carcasses and wood chips from gardening. The pile has decomposition phases that were considered active degradation (before aeration) and mature compost (after aeration). Pile aeration was achieved by the mechanical turning of the material after 50 to 60 days of composting. The temperature of the pile was monitored at five different points (four sides and one center). The average temperature of the pile at the time of collection was 50 °C.

#### Microbial isolation

Compost samples (10 g) were collected from the piles and diluted in 90 mL of sterile water. Serial dilutions (−2, −4 and −6) were performed and spread on agar plates of two selective halophilic media: JCM nº 377 medium (10% [w/v] NaCl, 0.5% [w/v] casamino acids, 0.5% [w/v] yeast extract, 0.2% [w/v] KCl, 0.3% [w/v] sodium citrate, 2% [w/v] MgSO_4_•7H_2_O, 0.036% [w/v] FeCl_2_ 4H_2_O, 0.00036% [w/v] MnCl_2_ 4H_2_O and 2% [w/v] agar, pH 7.2) and YPC medium (0.5% [w/v] yeast extract, 0.1% [w/v] peptone and 0.1% [w/v] casamino acids with 60% [v/v] of salt water solution 24% [w/v] NaCl, 3% [w/v] MgCl_2_ 6H_2_O, 3.5% [w/v] MgSO_4_•7H_2_O, 0.1% [w/v] KCl, 20 mM Tris HCl pH 7.5 and 3 mM CaCl_2_) and incubated at 30, 37 and 42 °C. After 24 or 48 h, the colonies were selected and transferred separately to obtain purified colonies.

### Screening of secreted extracellular hydrolytic activities

Enzymatic agar plate assays were performed to detect the presence of extracellular hydrolases. All media were adjusted to pH 7.3, and NaCl was added to obtain a salt concentration in the range of 0–4 M. The composition of the media used is described below.

#### Determination of extracellular amylase activity

Amylolytic activity on plates was determined qualitatively using a previously described method (Pascon *et al.*, 2011), which was modified for halophilic microorganisms by adding NaCl in the medium. After incubation at 37 °C for 5 days, the plates were exposed to iodine crystals for 5 min to reveal the starch degradation zone that indicates amylolytic activity.

#### Determination of extracellular protease activity

The cultures were screened in JCM nº 377 medium and YPC medium supplemented with 1% skim milk for the determination of protein hydrolytic activity. Clear zones around the colonies after 7 days were taken as evidence of proteolytic activity.

#### Determination of extracellular lipase activity

Lipase production by the isolated microorganisms was evaluated in nutrient agar tributyrin medium (NAT), which consisted of 1.3% nutrient broth, 1% tributyrin and 2% agar (Ben-Gigirey *et al.*, 2000). After incubation at 37 °C for 7 days, the hydrolytic zones around the bacterial colonies were considered an indication of lipase production.

#### Determination of extracellular cellulase activity

Cellulase activity was screened on a solid medium containing carboxymethyl cellulose (CMC) (Rohban *et al.*, 2009). After incubation at 37 °C for 7 days, the plates were flooded with 0.1% Congo red solution. The clear zone around colonies indicated cellulolytic activity.

### Screening of polyhydroxyalkanoates and exopolysaccharides

#### Detection of polyhydroxyalkanoate (PHA)-producing microorganisms

The isolates were evaluated in mineral medium (Schlegel *et al.*, 1970) with 2.5 M NaCl and containing glucose, xylose or octanoic acid as the carbon source. Glucose is known to be a carbon source for the production of short-chain-length PHAs, whereas octanoic acid produces medium-chain-length PHAs. Sugarcane bagasse contains xylose, and its excess is a promising substrate for producing by-products, such as second-generation bioethanol and PHAs (Lopes *et al.*, 2009). After 24 h of incubation (30 °C), the isolated strains were evaluated for their ability to grow on these carbon sources; the isolates were stained with Sudan Black B after 72 h to verify their potential to produce PHAs*.*

#### Detection of exopolysaccharide (EPS) producers

The isolates were cultivated in Bushnell Haas Salt Medium (50 mL) containing 2.5 M NaCl with glycerol as sole the carbon source for the microbial growth. After incubation for 5 days at 30 °C in a rotary shaker (150 rpm), the cultures were centrifuged at 8,200 × *g* for 15 min (4 °C). The emulsification index (E24) of the supernatant was evaluated according to the method described by Fleck *et al.* (2000) using hexadecane as a hydrophobic model compound. The chemical composition of EPSs precipitated from the supernatant with ethanol up to 70% was dialyzed against pure water, and carbohydrates, proteins and uronic acids were quantified in the retained high molecular weight fraction, as reported (Tanasupawat *et al.*, 2010).

### Bacterial identification

#### Mass spectrometry

The isolated microorganisms were treated with ethanol/formic acid for content extraction, following a previously described protocol (Pascon *et al.*, 2011). Measurements were conducted with a Microflex LT mass spectrometer (Bruker Daltonics) using FlexControl software (version 3.0, Bruker Daltonics) in the positive linear mode (laser frequency, 20 Hz; ion source 1 voltage, 20 kV; ion source 2 voltage, 18.6 kV; lens voltage, 7.5 kV; mass range, 2000 to 20 000 Da). For each spectrum, 240 shots in 50-shot steps from different positions of the target spot (automatic mode) were collected and analyzed. The spectra were internally calibrated using *Escherichia coli* ribosomal proteins. The raw spectra were imported into the BioTyper software (version 2.0, Bruker Daltonics) and processed by standard pattern matching with default settings; the results were reported in a ranking table.

#### Amplification and sequencing of 16S rRNA gene fragment

DNA (30–50 ng) from each strain was incubated in a 50-μL reaction mixture containing 2 mM MgCl_2_, 200 μM dNTPs, 0.3 μM universal primer 27f (5-AGAGTTGATCCTGGCTCAG-3), 0.3 μM 1525r (5-AAGGAGGTGWTCCARCC-3) and 2 U *Taq* DNA polymerase (Invitrogen) in the recommended buffer. Amplification was performed in a Veriti 96 well Thermal Cycler (Applied Biosystems) with an initial temperature at 94 °C for 2 min, 30 cycles at 94 °C for 1 min, 55 °C for 1 min and 72 °C for 3 min. A final extension at 72 °C was included for 10 min. The PCR products were purified with a GFX PCR DNA and gel band purification kit (GE Healthcare), and the sequence analysis was performed using a 3500 Genetic Analyzer Sequencer (Applied Biosystems). Subsequently, 5.0 μL purified PCR product was mixed with 4.0 μL of BigDye v. 3.1 (Applied Biosystems) and 1.0 μL sequencing primer (0.5 μmol). The primers used in the sequencing reactions were 27f (Dojka *et al.*, 1998), 782r (5ACCAGGGTATCTAATCCTGT3) (Chun and Goodfellow, 1995) and 1401r (5CGGTGTGTACAAGACC C3) (Nübel *et al.*, 1996). The sequencing program consisted of 25 cycles at 95 °C for 20 s, 50 °C for 15 s and 60 °C for 60 s. The 16S rRNA gene sequence of all the analyzed strains was compared to bacterial sequences deposited in GenBank. Sequences with similarity were retrieved, and the consensus sequences were aligned using CLUSTALW with MEGA 5.05. EzTaxon tools (http://147.47.212.35:8080/) were further employed to confirm the similarities, and phylogenetic trees were constructed based on neighbor-joining, maximum-likelihood and maximum-parsimony methods. The resulting tree topologies were evaluated by a bootstrap analysis based on 1000 replicates.

### Nucleotide sequence accession numbers

The sequenced strains SR5-6, SR5-7, SR5-12, YPC-6, YPC-8, YPC-11, YPC-13 and YPC-15 were deposited in GenBank under accession numbers JX154082, JX154083, JX154084, JX154085, JX154086, JX154087, JX154088 and JX154089, respectively.

## Results and Discussion

### Identification of halophilic strains

**Figure 2:**
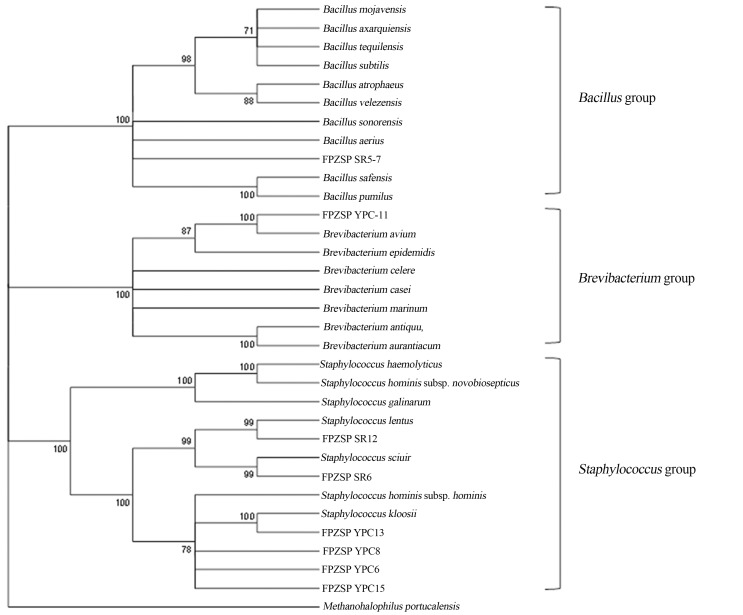
Phylogenetic tree showing the position of the halotolerant isolates, as based on a partial 16S rRNA gene sequence comparison obtained by neighbor-joining and maximum-likelihood trees. The nucleotide sequence accession numbers were deposited in GenBank, as described in Material and Methods.

The isolated bacteria were obtained from the composting process during the turning stage (60^th^ day). Eight out of eleven halophilic isolates in 2.5 M NaCl from the composting process were subjected to a MALDI-TOF mass spectrometry analysis, which indicated that the genera of all isolates were Gram positive, which was confirmed by Gram staining. These procedures ensured and confirmed the purity of the isolates. The 16S rRNA gene sequences of eight strains (> than 1300 bp) were compared with those previously deposited in GenBank. The neighbor-joining and maximum-likelihood trees showed the taxonomic position of these strains, which were affiliated with *Bacillus*, *Staphylococcus* and *Brevibacterium* genera (Figure 2)*.*

Strain SR5-7 showed high 16S rRNA gene sequence similarity to *Bacillus* when compared with the 184 different species of this genus. However, based on the similarity matrix of the 16S rRNA gene, this isolate did not show 100% similarity with any of the species already reported. The species of *Bacillus* described as halophilic to date are as follows: *B. hemicentroti* (Chen *et al.*, 2011); *B. humanensis* (Chen *et al.*, 2011); *B. xianensis* (Sanchez-Porro *et al.*, 2003; Schlegel *et al.*, 1970); *B. alkaliphilic* (Zhang *et al.*, 2012); *B. halochares* (Pappa *et al.*, 2010); *B. chungangensis* (Cho *et al.*, 2010) and *B. subtilis* (Takenaka *et al.*, 2011). Thus, the possibility that a new species of halophilic *Bacillus* was isolated from a compost process is noteworthy.

The YPC-11 strain was identified as *Brevibacterium avium* (100% similarity). An EzTaxon analysis confirmed that this strain shared 100% 16S rRNA gene sequence similarity with *B. avium* and 99.97% with *Brevibacterium epidermidis*, the only halotolerant bacterium (Nagata and Wang, 2005) described in the genus *Brevibacterium*.

Strains SR5-12, SR5-6, YPC-6, YPC-8, YPC-13 and YPC-15 were classified as members of the *Staphylococcus* genus. The isolates SR5-12 and SR5-6 showed 100% 16S rRNA gene sequence similarity with *S. lentus* and *S. sciuri*, respectively, a result that was confirmed by EzTaxon. However, to date, these species have not been described as high salt concentration-tolerant bacteria, with the only species of *Staphylococcus* known as halophilic being *Staphylococcus equorum* (Essghaier *et al.*, 2009).

All the selected isolates were deposited at the São Paulo Zoo Park Culture Collection (SPZSP-CCol).

### Salt tolerance and growth of halophilic isolates

All the bacteria isolated in 2.5 M NaCl were tested for their ability to grow at different salt concentrations. A slowing of bacterial growth was observed in the presence of high salt concentrations, as indicated by the time (in days) required for detecting the presence of bacteria in the culture medium (Table 1). *Staphylococcus* strains SR5-12, YPC-6, SR5-6 and YPC-8 showed similar growth behavior from 0 to 4.0 M NaCl. Strain YPC-13 had the slowest growth at high salinity, and strain YPC-15 grew preferentially at 2.5 M NaCl or higher. *Bacillus* strains SR5-7 and YPC-11 (affiliated with *B. avium*) exhibited a preference for growing in a culture medium containing 0.5 to 2.0 M NaCl but failed to grow in 4.0 M NaCl. It is important to note that although all of these bacteria tolerated high salinities (2.5 M NaCl or higher), they are not strictly halophilic bacteria. According to Kushner (1978), bacteria that are able to grow in the absence of salt as well as in the presence of relatively high salt concentrations are designated halotolerant or extremely halotolerant if growth extends above 2.5 M. Based on this classification, seven out of the eight isolated microorganisms isolated from composting process were halotolerant or extremely halotolerant. It should be noted that the salt requirement and tolerance of many species vary according to the growth conditions, such as temperature and medium composition.

**Table 1:** Different types of hydrolytic activities, amylase (A), cellulase (C), lipase (L) and protease (P), found in the eight strains isolated from OCPU at the 0 – 4.0 M NaCl concentration range.

	SR5-6 (*Staphylococcus* sp.)		SR5-7 (*Bacillus* sp.)		SR5-12 (*S. lentus*)		YPC-6 (*Staphylococcus* sp*.*)		YPC-8 (*Staphylococcus* sp*.*)		YPC-11 (*B. avium*)		YPC-13 (*Staphylococcus* sp.)		YPC-15 (*Staphylococcus* sp.)	
								
[NaCl]	Days	Hydrolase	Days	Hydrolase	Days	Hydrolase	Days	Hydrolase	Days	Hydrolase	Days	Hydrolase	Days	Hydrolase	Days	Hydrolase
0 M	1	Nd	1	L, P	1	L, P	1	Nd	2	L	2	A, C	2	Nd	4	A, C, L
0.5 M	1	L	1	L, P	1	L	1	Nd	2	L	2	A, C	2	Nd	0	Nd
1.0 M	1	Nd	1	L	1	L	1	Nd	2	L	2	A, C	2	Nd	0	Nd
1.5 M	1	Nd	1	L	1	Nd	1	Nd	2	L	2	A, C	2	Nd	0	Nd
2.0 M	1	Nd	1	L	1	Nd	1	Nd	2	L	3	A, C	3	Nd	1	A, C
2.5 M	1	Nd	2	Nd	2	Nd	2	Nd	2	L	4	A, C	7	Nd	2	A, C
3.0 M	2	Nd	2	Nd	2	Nd	2	Nd	2	Nd	7	A, C	7	Nd	2	A, C
3.5 M	4	Nd	2	Nd	2	Nd	3	Nd	3	Nd	21	A, C	21	Nd	3	A, C
4.0 M	7	Nd	-	Nd	4	Nd	4	Nd	4	Nd	21	A, C	21	Nd	3	A, C

The assays were performed as described in Material and Methods. Days: Time in days that visible growth was observed, considering zero to be the day of inoculation. (Nd): Hydrolytic activity not detected.

Several bacteria of *Bacillus*, *Halobacillus* and *Staphylococcus* have been found in saline environments, such as Salt Plains National Wildlife Refuge, Great Salt Plains of Oklahoma, a Bolivian hypersaline lake, deep-sea sediments and tropical marine sediments (Ventosa *et al.*, 1998). Some species of *Bacillus* sp. are salt tolerant and are important degraders of organic pollutants. Examples include *Bacillus cereus*, which degrades 1,3-dichlorobenzene derivatives from town-gas industrial influent (Wang *et al.*, 2003), and *Bacillus subtilis*, which degrades p-aminobenzene from textile industry wastewater (Zissi *et al.*, 1997).

### Hydrolytic activities of halotolerant isolates

The SR5-6, SR5-7, SR5-12, YPC-8, YPC-11 and YPC-15 strains were found to be moderate halophilic microorganisms and showed combined cellulolytic, amylolytic, lipolytic and proteolytic activities (Table 1). These strains have potential biotechnological applications with respect to their ability to produce different hydrolases (Rohban *et al.*, 2009). In contrast, no hydrolytic activity was observed for YPC-6 and YPC-13.

Only YPC-11 (affiliated with *B. avium*) presented amylase and cellulase hydrolytic activities from 0 to 4 M NaCl. Members of the genus *Bacillus* are well known enzyme producers, and many industrial processes utilize species belonging to this genus for the commercial production of enzymes (Vasconcellos *et al.*, 2011). The strain SR5-7 (affiliated with *Bacillus*) produced lipase and protease in 2.0 M and 0.5 M NaCl, respectively. It is interesting to note that the lipase producers reported thus far are limited to representatives of the genera *Salinivibrio*, *Halomonas* and *Bacillus-Salibacillus* (Sanchez-Porro *et al.*, 2003).

### Polyhydroxyalkanoate (PHA) producers

All the isolates were evaluated using a medium with nitrogen limitation and different carbon sources (Table 2). The isolates grew better with glucose as the sole carbon source compared to xylose and octanoic acid. The isolates YPC-13 (affiliated with *Bacillus* sp.), SR5-7 (affiliated with *Bacillus* sp.) and YPC-15 (affiliated with *Staphylococcus* sp.) accumulated PHAs in presence of octanoic acid, xylose and glucose, respectively. The genus *Bacillus* is known as a producer of PHAs (Lopes *et al.*, 2009), and *Staphylococcus epidermidis*, which was isolated from sesame oil, presented the ability to produce poly-3-hydroxybutyrate (Wong *et al.*, 2000). The strain YPC-11 (affiliated with *B. avium*) was detected as a potential producer of biopolymers using octanoic acid and xylose. This result is in accordance with the previous observation that *Brevibacterium casei* (SRKP2 strain) could produce PHAs in a medium containing dairy industrial waste, yeast extract and sea water (Pandian *et al.*, 2009). Halotolerant microbes are important for the biotechnology industry due to their advantages for use in sterilization processes and the control of contaminants; the PHA-producing halophilic microorganisms have recently been reviewed (Poli *et al.*, 2011). The production of PHAs using xylose is an alternative strategy to produce economically competitive PHAs using agro-industrial products such as sugarcane molasses and bagasse (Gomez *et al.*, 2012).

**Table 2:** Analysis of isolates for the ability to produce EPSs in mineral medium and PHAs using different carbon sources in the presence of 2.5 M NaCl.

	SR-6 (*Staphylococcus* sp.)	SR-7 (*Bacillus* sp.)	SR-12 (*S. lentus*)	YPC-6 (*Staphylococcus* sp.)	YPC-8 (*Staphylococcus* sp.)	YPC-11 (*B. avium*)	YPC-13 (*Staphylococcus* sp.)	YPC-15 (*Staphylococcus* sp.)
EPS (E24)	58%	50%	51%	53%	50%	55%	55%	55%
PHA	Nd	Xylose (+)	Nd	Nd	Nd	Xylose (+) Octanoic acid (+)	Octanoic acid (+)	Glucose (+)

(E24): Emulsification indexes of the isolates in a mineral medium containing 2.5 M NaCl, as described in Materials and Methods.

(+): indicates that PHAs were detected when using the indicated carbon source.

(Nd): PHA production not detected.

### EPS production and emulsification potential

Microbial exopolymers (EPSs) correspond to compounds produced by microorganisms to solubilize essential nutrients for their survival or to promote their adherence onto surfaces (Ron and Rosenber, 2002). The use of glycerol as a sole carbon source and 2.5 M NaCl resulted in EPS values up to 60% of the emulsification index (E24) of hexadecane (Table 2). A colorimetric analysis showed that the biosurfactant produced by the evaluated halotolerant strains were mainly composed of carbohydrates (95%) but also contained proteins (0.5%) and uronic acids (4.5%) in their composition. A similar EPS composition was also reported in halophilic Archaea strains (Poli *et al.*, 2011).

## Conclusion

Screens for halotolerant or halophilic microorganisms in non-saline environments are scarce as is the detection of extracellular enzymes. This study found eight isolates from an organic residue composting process that showed the ability to tolerate a wide range of salinity. Some of these strains presented combined hydrolytic ability in the presence of NaCl. The possibility of these microorganisms, particularly YPC-11 (affiliated with *B. avium*), to produce EPSs and PHAs in the presence of 2.5 M NaCl can offer new biotechnological and bioremediation perspectives for the treatment of oilfield wastes as well as in MEOR (microbial-enhanced oil recovery) processes. The performance of the halotolerant isolates in the present work were not compared to other already known and classic halophilic microorganisms, and this should be performed in future work.

## References

[B01] Ben-Gigirey B, de Sousa JMVB, Villa TG (2000). Characterization of biogenic amine-producing
*Stenotrophomonas maltophilia* strains isolated from
white muscle of fresh and frozen albacore tuna. Int J Food Microbiol.

[B02] Chen YG, Hao DF, Chen QH (2011). Bacillus hunanensis sp nov., a slightly halophilic bacterium
isolated from non-saline forest soil. Antonie Van Leeuwenhoek Int J Genet Mol Microbiol.

[B03] Chen YG, Zhang YQ, He JW (2011). *Bacillus hemicentroti* sp. nov., a moderate
halophile isolated from a sea urchin. Int J Syst Evol Microbiol.

[B04] Cho SL, Jung MY, Park MH (2010). *Bacillus chungangensis* sp. nov., a halophilic
species isolated from sea sand. Int J Syst Evol Microbiol.

[B05] Chun J, Goodfellow M (1995). A phylogenetic analysis of the genus *Norcadia*
with 16S rDNA gene sequences. Int J Syst Bacteriol.

[B06] Dojka MA, Hugenholtz P, Haack SK (1998). Microbial diversity in a hydrocarbon-and
chlorinated-solvent-contaminated aquifer undergoing intrinsic
bioremediation. Appl Environ Microbiol.

[B07] Enache M, Kamekura M (2010). Hydrolytic enzymes of halophilic microorganisms and their
economic values. Rom J Biochem.

[B08] Essghaier B, Fardeau ML, Cayol JL (2009). Biological control of grey mould in strawberry fruits by
halophilic bacteria. J Appl Microbiol.

[B09] Fleck LC, Bicca FC, Ayub MAZ (2000). Physiological aspects of hydrocarbon emulsification, metal
resistance and DNA profile of biodegrading bacteria isolated from oil
polluted sites. Biotechnol Lett.

[B10] Fuciños P, Gonzalez R, Atanes E (2012). Lipases and esterases from extremophiles: overview and case
example of the production and purification of an esterase from
*Thermus thermophilus* HB27. Methods Mol Biol.

[B11] Gomez J, Mendez B, Nikel P, Petre M (2012). Making Green polymers even greener: Towards sustainable
production of polyhydroxyalkanoates from agroindustrial
by-products. Adv Appl Biotechnol.

[B12] Gupta R, Beg QK, Lorenz P (2002). Bacterial alkaline proteases: molecular approaches and industrial
applications. Appl Microbiol Biotechnol.

[B13] Kushner DJ (1978). Life in High Salt and Solute Concentrations: Halophilic
Bacteria.

[B14] Legat A, Gruber C, Zangger K (2010). Identification of polyhydroxyalkanoates in Halococcus and other
haloarchaeal species. Appl Microbiol Biotechnol.

[B15] Li X, Yu HY (2011). Extracellular production of beta-amylase by a halophilic isolate,
*Halobacillus* sp. LY9. J Ind Microbiol Biotechnol.

[B16] Litchfield CD (2012). Potential for industrial products from the halophilic
Archaea. J Ind Microbiol Biotechnol.

[B17] Lopes MSG, Rocha RCS, Zanotto SP (2009). Screening of bacteria to produce polyhydroxyalkanoates from
xylose. World J Microbiol Biotechnol.

[B18] Martins LF, Antunes LP, Pascon RC (2013). Metagenomic analysis of a tropical composting operation at the
São Paulo zoo park reveals diversity of biomass degradation functions and
organisms. PLoS One.

[B19] Nagata S, Wang CX (2005). Efficient utilization of ectoine by halophilic Brevibacterium
species and Escherichia coli subjected to osmotic down shock. J Biosci Bioeng.

[B20] Nübel U, Engelen B, Felske A (1996). Sequence heterogeneities of genes encoding 16S rRNAs in
Paenibacillus polymyxadetected by temperature gradient gel
electrophoresis. J Bacteriol.

[B21] Pandian SRK, Deepak V, Kalishwaralal K (2009). Synthesis of PHB nanoparticles from optimized medium utilizing
dairy industrial waste using *Brevibacterium casei* SRKP2: A
green chemistry approach. Colloids Surf B-Biointerfaces.

[B22] Pappa A, Sanchez-Porro C, Lazoura P (2010). *Bacillus halochares* sp. nov., a halophilic
bacterium isolated from a solar saltern. Int J Syst Evol Microbiol.

[B23] Partanen P, Hultman J, Paulin L (2010). Bacterial diversity at different stages of the composting
process. BMC Microbiol.

[B24] Pascon RC, Bergamo RF, Spinelli RX (2011). Amylolytic Microorganism from São Paulo Zoo Composting:
Isolation, Identification, and Amylase Production. Enzym Res.

[B25] Poli A, Di Donato P, Abbamondi GR (2011). Synthesis, Production, and Biotechnological Applications of
Exopolysaccharides and Polyhydroxyalkanoates by Archaea. Archaea.

[B26] Prakash B, Vidyasagar M, Madhukumar MS (2009). Production, purification, and characterization of two extremely
halotolerant, thermostable, and alkali-stable alpha-amylases from
*Chromohalobacter* sp TVSP 101. Process Biochem.

[B27] Rohban R, Amoozegar MA, Ventosa A (2009). Screening and isolation of halophilic bacteria producing
extracellular hydrolases from Howz Soltan Lake, Iran. J Ind Microbiol Biotechnol.

[B28] Ron EZ, Rosenberg E (2002). Biosurfactants and oil bioremediation. Curr Opin Biotechnol.

[B29] Sanchez-Porro C, Martin S, Mellado E, Ventosa A (2003). Diversity of moderately halophilic bacteria producing
extracellular hydrolytic enzymes. J Appl Microbiol.

[B30] Schlegel HG, Lafferty R, Krauss I (1970). Isolation of mutants not accumulating poly-beta-hydroxybutyric
acid. Arch Für Mikrobiol.

[B31] Schloss PD, Hay AG, Wilson DB (2003). Tracking temporal changes of bacterial community fingerprints
during the initial stages of composting. FEMS Microbiol Ecol.

[B32] Takenaka S, Yoshida N, Yoshida K (2011). Molecular cloning and sequence analysis of two distinct
halotolerant extracellular proteases from *Bacillus subtilis*
FP-133. Biosci Biotechnol Biochem.

[B33] Tanasupawat S, Chamroensaksri N, Kudo T (2010). Identification of moderately halophilic bacteria from Thai
fermented fish (pla-ra) and proposal of *Virgibacillus
siamensis* sp. nov. J Gen Appl Microbiol.

[B34] Vasconcellos SP, Dellagnezze BM, Wieland A (2011). The potential for hydrocarbon biodegradation and production of
extracellular polymeric substances by aerobic bacteria isolated from a
Brazilian petroleum reservoir. World J Microbiol Biotechnol.

[B35] Ventosa A, Nieto JJ (1995). Biotecnological applications and potentialities of halophilic
microrganisms. World J Microbiol Biotechnol.

[B36] Ventosa A, Nieto JJ, Oren A (1998). Biology of moderately halophilic aerobic bacteria. Microbiol Mol Biol Rev.

[B37] Voget S, Steele HL, Streit WR (2006). Characterization of a metagenome-derived halotolerant
cellulase. J Biotechnol.

[B38] Wang L, Zhou Q, Zhang BS (2003). The biodegradation of 1,3-dichlorobenzene by an adapted strain
*Bacillus cereus* PF-11 derived from town-gas industrial
effluent. J Environ Sci Health Part A.

[B39] Wong AL, Chua H, Yu pH (2000). Microbial production of polyhydroxyalkanoates by bacteria
isolated from oil wastes. Appl Biochem Biotechnol.

[B40] Zhang GM, Li SY, Xue YF (2012). Effects of salts on activity of halophilic cellulase with
glucomannanase activity isolated from alkaliphilic and halophilic
*Bacillus* sp. BG-CS10. Extremophiles.

[B41] Zissi U, Lyberatos G, Pavlou S (1997). Biodegradation of p-aminoazobenzene by *Bacillus
subtilis* under aerobic conditions. J Ind Microbiol Biotechnol.

